# Mechanisms of Mitochondrial ROS Production in Assisted Reproduction: The Known, the Unknown, and the Intriguing

**DOI:** 10.3390/antiox9100933

**Published:** 2020-09-29

**Authors:** James N. Cobley

**Affiliations:** Redox Biology Group, Institute for Health Sciences, University of the Highlands and Islands, Old Perth Road, Inverness IV2 3JH, UK; james.cobley@uhi.ac.uk

**Keywords:** mitochondria, oxidative stress, reactive oxygen species, assisted reproduction technology, development, oocyte

## Abstract

The consensus that assisted reproduction technologies (ART), like in vitro fertilization, to induce oxidative stress (i.e., the known) belies how oocyte/zygote mitochondria—a major presumptive oxidative stressor—produce reactive oxygen species (ROS) with ART being unknown. Unravelling how oocyte/zygote mitochondria produce ROS is important for disambiguating the molecular basis of ART-induced oxidative stress and, therefore, to rationally target it (e.g., using site-specific mitochondria-targeted antioxidants). I review the known mechanisms of ROS production in somatic mitochondria to critique how oocyte/zygote mitochondria may produce ROS (i.e., the unknown). Several plausible site- and mode-defined mitochondrial ROS production mechanisms in ART are proposed. For example, complex I catalyzed reverse electron transfer-mediated ROS production is conceivable when oocytes are initially extracted due to at least a 10% increase in molecular dioxygen exposure (i.e., the intriguing). To address the term oxidative stress being used without recourse to the underlying chemistry, I use the species-specific spectrum of biologically feasible reactions to define plausible oxidative stress mechanisms in ART. Intriguingly, mitochondrial ROS-derived redox signals could regulate embryonic development (i.e., their production could be beneficial). Their potential beneficial role raises the clinical challenge of attenuating oxidative damage while simultaneously preserving redox signaling. This discourse sets the stage to unravel how mitochondria produce ROS in ART, and their biological roles from oxidative damage to redox signaling.

## 1. Mitochondrial ROS, Oxidative Stress, and Assisted Reproduction: An Introduction 

From a historical perspective, the ability of mitochondria to produce superoxide—a key reactive oxygen species (ROS, see Box 1)—has been known since 1966 [1]. Two years later, Auerbach and Brinster [2] found that: exposing mouse zygotes to atmospheric ground-state molecular dioxygen (O_2_) levels (i.e., 21% O_2_) underlies the so-called “2-cell block” to embryo culture, wherein zygotes fail to progress to the 4-cell stage or exhibit severely delayed development. Importantly, lowering [O_2_] by 16% from 21 to 5%, a plausible in utero value, overcome the 2-cell block. A decade later, their findings would have important repercussions for assisted reproduction technologies (ART), like in vitro fertilization (IVF), when the first “test-tube” baby was born in 1978 [2]. In the subsequent ~40 years: (1) ART has become invaluable for treating infertility, which currently affects 15% of couples worldwide [3]; (2) Helmut Sies introduced the term oxidative stress [4] and the biochemistry of key species like superoxide is now well-understood [5]; (3) we now understand much of the mechanistic basis of oxidative phosphorylation (OXPHOS) and mitochondrial superoxide production [6] (e.g., beyond complex I and III, we now know that over 10 enzymes can produce ROS [7]); and (4) a nuanced view of the biological role of ROS has emerged (e.g., as opposed to being purely damaging agents, ROS are now considered to play beneficial biological roles [8]). In 2020, it is, therefore, surprising that little can be stated with any great confidence about how mitochondria produce ROS in ART. The “known” extends little further than: (1) oocyte/zygote mitochondria produce ROS [9,10,11]; (2) oocyte/zygote mitochondria derived from older females tend to produce more ROS [12,13,14]; and (3), in an ageing setting at least, certain mitochondria-targeted antioxidants seem protective [14,15]. Moreover, the wider chemical biology of ART-induced oxidative stress is unclear. Taking the 2-cell block as an example, mechanistic understanding is fragmentary, because chemically-defined source-target relationships are lacking. In the clinic, insufficient mechanistic understanding means that how culturing embryos at 5% O_2_ improves live birth rates by ~13% is unclear [16]. Unravelling the underlying mechanisms holds promise for increasing live birth rates by rationally targeting oxidative stress. 

Against this backdrop, I critique how somatic mitochondria produce ROS (“the known”; Section 2) as a resource to address “the unknown” mechanisms of mitochondrial ROS production in ART (Section 3). Extending the knowns (e.g., the sites and modes will evolve with time) and making plausible experimentally testable hypotheses about the unknowns (e.g., novel oocyte extraction-induced ROS production mechanisms) advances current understanding. Finally, I consider “the intriguing” potential functional roles of mitochondrial ROS production in ART (Section 4). To do so, I present a chemically-defined framework to explain how mitochondrial ROS may cause oxidative stress. Importantly, the paradigm shifting ability of mitochondrial ROS-derived redox signals to regulate development is considered (i.e., their production could be beneficial [17]). Before proceeding, the present discourse focuses on mitochondria, because of their strategic importance [18], complex redox biology (i.e., there are over 10 differentially regulated sites of ROS production in mitochondria), and the mechanisms for how cytosolic ROS sources, like NADPH oxidase enzymes or xanthine oxidase, for example, produce ROS are well understood [19]. Section 4 remains relevant to any reader interested in understanding how cytosolic ROS cause oxidative stress. For the purposes of brevity, the present review is delimited to mammals, complex I-III, and oocyte/zygote mitochondria (sperm are considered elsewhere [20]).

Box 1 **Oxygen, ROS and Oxidative stress: A primer.**

**Oxygen**: Ground state molecular dioxygen (O_2_) is a free radical—a molecule capable of independent existence with at least one unpaired electron—because it contains two unpaired electrons with parallel spins [8]. Parallel spins (i.e., ↑↑) make aerobic life possible by spin-restricting the ability of O_2_ to react appreciably with most spin paired (i.e., ↑↓) biomolecules [21,22,23]. The importance of spin restriction is underlined by the ability of singlet oxygen (∆^1^O_2_, an electronically excited species) to oxidize several biomolecules, because the excitation energy spin pairs (i.e., ↑↓) the two electrons [24]. ∆^1^O_2_ is an excellent example of a non-radical species with greater chemical reactivity than the parent radical. Spin restriction limits O_2_ to single electron transfers [8]. Single electron transfers are essential in mitochondria, wherein cytochrome c oxidase (Complex IV) splits O_2_ to atomic oxygen, before reducing it to form water (H_2_O) without releasing catalytic radical intermediates [25].

**ROS**: The umbrella term reactive oxygen species (ROS) encompasses the free radicals superoxide anion (O_2_^●−^) and hydroxyl radical (^•^OH), as well as the non-radical hydrogen peroxide (H_2_O_2_). Their interrelationship is summarized below:

O_2_ → O_2_^●−^ → H_2_O_2_→ ^●^OH ↔ H_2_O

The chemistry and metabolism of each species are discussed elsewhere [8,26,27,28]. Relevant points are threefold. First, their chemical reactivity differs by orders of magnitude. For example, OH oxidizes DNA a billion times faster than O_2_^●−^ or H_2_O_2_. Second, the biochemistry of O_2_^●−^ and H_2_O_2_ is selective—they react rapidly with a few strategically important biomolecules—whereas OH is chemically promiscuous. Third, their intracellular concentrations seldom rise above nanomolar (i.e., 10^−9^ M) levels due to efficient metabolism or diffusion-controlled reactivity in the case of ^●^OH.

**Oxidative stress**: Helmut Sies first coined the term oxidative stress in 1985 [4]; his subsequent work defines oxidative stress as increased oxidative damage and/or disrupted redox signaling [29,30,31]. Redox signaling refers to the ability of ROS to transduce intracellular signals, which is a central tenet of the redox code proposed by Sies and Jones [32]. Oxidative stress is a frequently used but seldom chemically qualified term [33]. That is, oxidative stress confers no useful mechanistic information unless the chemical details are disclosed [29,34]. While unravelling the underlying chemistry is a challenging task [35], it is required to rationally target oxidative stress. For example, insufficient mechanistic understanding played a key part in the failure of vitamin E and C to treat disease—they seem to have little ability to react with relevant species at the relevant time and place [8,36].

## 2. Mechanisms of Mitochondrial ROS Production: The Known

### 2.1. The Major and Minor Mitochondiral Electron Pathways

**Figure 1:**
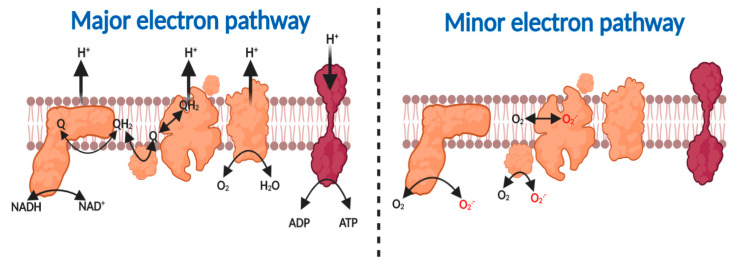
Major and minor electron pathways in the mitochondrial respiratory chain. Major pathway (**left** to **right**). Complex I oxidizes NADH to NAD^+^ to reduce ubiquinone (Q) to ubiquinol (QH_2_). Complex II oxidizes succinate to fumarate to produce QH_2_. Complex III oxidizes QH_2_ to reduce cytochrome c. Reduced cytochrome c is then oxidized by complex IV to reduce O_2_ to H_2_O. The proton motive force generated by complex I, III and IV is harnessed by Complex V to synthesize ATP. Minor pathway left to right. Substrate-derived electrons can reduce O_2_ to O_2_^●−^ at complex I, II and III.

The major fate of substrate-derived electrons—free radicals—tunneling (a quantum mechanical property that enables electrons to penetrate a potential energy barrier without further input energy), according to the principles of quantum mechanics, through the redox centers nestled within the mitochondrial respiratory complexes is to reduce O_2_—a diradical—to H_2_O (reaction 1, see Figure 1) [21,25,37,38,39,40]. The free energy associated with the thermodynamically favorable electron transfer from NADH (E^o^’ = −340 mV) or FADH (E^o^’ = +31 mV) via ubiquinol (E^o^’ = +45 mV) to O_2_ (E^o^’ = +840 mV) is used by complex I, III and IV to pump protons, to create an electrochemical proton motive force (Δ*p*), comprising a membrane potential (ΔΨ_m_~−150–200 mV) and pH component (ΔpH = ~0.8), across the inner mitochondrial membrane [6,41,42,43]. Δ*p* enables OXPHOS by forcing the F_1_-F_o_ ATP synthase (i.e., complex V) to synthesize, as opposed to hydrolyze, ATP (reaction 2) [44,45,46]. A minor fate of substrate-derived electrons in the respiratory chain is to support the univalent reduction of O_2_ to superoxide at complex I, II and III (reaction 3, see Box 1 and Figure 1) [47].

**reaction 1:** O_2_ + 8H^+^ +4 e –> 2H_2_O + Δ*p* (4H+ pumped)

**reaction 2:** ADP + Pi + Δ*p* <–> ATP

**reaction 3:** e + O_2_ –> O_2_^−^ (superoxide)

### 2.2. How Mitochondria Produce Superoxide

The interested reader is referred to Murphy’s [47] classic account for a comprehensive overview of the field. At first glance, the thermodynamics of reaction 3 (E = −160 mV at pH 7) would appear to restrict superoxide production to highly reducing electron donors. When the Nernst equation is used to compute E^o’^ at plausible O_2_ (~3–30 µM) and superoxide (~100–200 pM), levels values of 150–230 mV are obtained [47]. Many thermodynamically competent NADH (E^o^’ = −340 mV), FADH (E^o^’ = +31 mV) or ubiquinol (E^o^’ = +45 mV) linked enzymes could, therefore, catalyze the univalent reduction of O_2_ to superoxide, provided a kinetic mechanism exists. Superoxide is typically produced via an outer sphere electron tunneling mechanism from the donor to acceptor [47,48,49]. The p*K*a of superoxide (4.8 [5]) means the bulk (~99%) exists as an anion (O_2_^●−^) as opposed to the hydroperoxyl radical (HO_2_^●^). In the mitochondrial matrix, the ratio of HO_2_^●^ to O_2_^●−^ is 1:1000 at pH 7.8. The reorganization energy for O_2_ to accept a single electron is simplified by the lack of proton transfer. Instead, outer sphere electron tunneling distance is key [37] (i.e., the rate decreases as the distance between donor and acceptor increases—explaining why ensconcing labile redox active iron-sulfur clusters deep within enzymes protects against facile superoxide production [50]). Mitochondrial superoxide production, for a given site, is set by the amount of the reduced enzyme in an O_2_ accessible form (E_RED_), the amount of O_2_, and the kinetics (*k*) of their second order bimolecular reaction [42,47,51]. The rate of total superoxide production over a set time interval can be calculated using Equation (1):

[O_2_^−^]/*t* = [O_2_] ∑(*k* [E_RED_]) (1)

where *k* is the weighted mean of the second order biomolecular reaction of all mitochondrial superoxide with O_2_ and [E_RED_] is the sum of their redox state. 

Despite Chance’s group clearly stating that 1–2% of O_2_ produces superoxide in isolated mitochondria under defined conditions [52,53], their finding has often erroneously been taken to mean that a fixed immutable percentage of O_2_ uptake supports superoxide production. The amount of mitochondrial [O_2_] that gives rise to superoxide varies over time according to the prevailing conditions. That is, no invariant immutable percentage exists [54]. Much of the superoxide produced by mitochondria is rapidly converted to H_2_O_2_ by manganese superoxide dismutase (MnSOD) [55,56,57]. Since superoxide, H_2_O_2_, and several other species (e.g., OH) co-exist in mitochondria (and in biological systems *per se*), the term Reactive Oxygen Species (ROS, see Box 1) is used. As Sies and Jones [58], as well as Halliwell and Gutteridge [8] remark, ROS is an umbrella term—no molecule called ROS actually exists! It is most rewarding to keep this in mind, when appraising oxidative stress mechanisms. Before considering ART, I define the key sites and proposed operational modes of mitochondrial superoxide production. 

### 2.3. Complex I: Forward Mode

**Figure 2:**
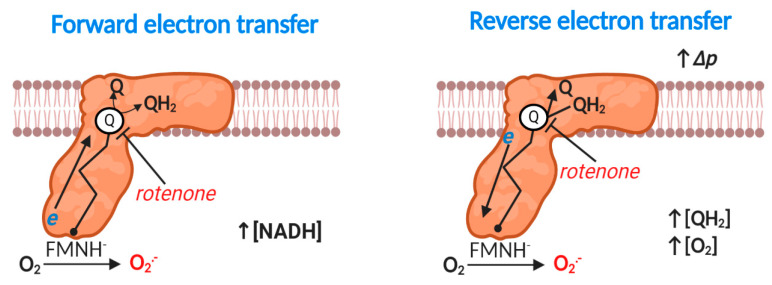
Superoxide production at mitochondrial complex I. Forward mode. At high NADH levels or when electron transfer between flavin mononucleotide radical (FMN) via seven iron sulfur (Fe-S) clusters to the Q reduction site stalls (e.g., rotenone inducible), reduced FMN transfers a single electron to O_2_ to produce O_2_^●−^. Reverse mode. When Δ*p* is near maximal and the Q pool is highly reduced, complex I produces substantial O_2_^●−^ by reverse electron transfer (RET). Rotenone blocks QH_2_ oxidation to inhibit RET-mediated O_2_^●−^ production. The exact site of O_2_^●−^ production is unclear (see text); the figure shows FMN catalyzed O_2_^●−^ production for the purposes of clarity. A flavin mononucleotide radical (FMN^●^) may also contribute. RET-mediated O_2_^●−^ production linearly depends on [O_2_].

Eukaryotic mitochondrial complex I (NADH: ubiquinone oxidoreductase) is a 1 megadalton, multi-subunit (14 core and 31 accessory), transmembrane enzyme responsible for coupling NADH oxidation to ubiquinone reduction and vectoral proton transfer by an unresolved spatially delocalized mechanism [59,60,61,62,63,64] (reaction 4). Hirst’s group [65,66] have established that: a partially reduced prosthetic flavin mononucleotide species (FMNH^-^) reacts with O_2_ to produce superoxide at complex I (reaction 5). Bound NAD^+^/NADH can, therefore, sterically occlude FMN-mediated superoxide production [65] (i.e., increasing the distance between donor and acceptor). Upstream bi and tetranuclear iron sulfur clusters can also control superoxide production by limiting FMNH^-^ (i.e., *t*E_RED_) lifetime (i.e., *t*E_RED_). An additional (i.e., bypassed in normal electron tunneling from FMN to the Q binding site) binuclear iron-sulfur cluster termed N1a may also modify superoxide production, potentially by sequestering electrons to decrease [FMNH^-^] or via a peptide bond gated switch [67,68]. When electron transfer stalls as occurs in the rotenone (a Q binding site inhibitor) inhibited complex, considerable superoxide production can occur (Figure 2). However, much superoxide can also emanate from tricarboxylic acid cycle (TCA) dehydrogenases at high NADH/NAD^+^ ratios (see Table 1), depending on the substrate supply [69].

**reaction 4:** NAD^+^ + Q + H^+^ (matrix) <–>NADH + QH_2_ + Δ*p*

**reaction 5:** FMNH^−^ + O_2_ –>FMNH + O_2_^●−^

### 2.4. Complex I: Reverse Electron Transfer

Energetically degenerate catalytic steps [62] render reaction 4 fully reversible, provided a sufficient thermodynamic driving force exists, which occurs when the free energy released from electron transfer is insufficient to pump protons against the prevailing Δ*p* [70] (Figure 2). Reverse electron transfer (RET) catalyzed superoxide production was discovered by Chance and co-workers in 1967 [71], and was considered irrelevant, until Murphy’s group showed that RET contributed to cardiac ischemia reperfusion injury (IRI) in 2013–2014 [72,73]. We now know that RET plays several important physiological (e.g., sleep, lifespan and O_2_ sensing) and pathological (e.g., in organ transplantation) roles [74,75,76,77,78,79,80]. Robb and colleagues [81] have identified the factors that govern RET: a highly reduced Q pool and near maximal Δ*p*. A near maximal Δ*p* necessitates low ATP synthesis (and limited activity of other Δ*p* consumers; e.g., the transhydrogenase or the adenine nucleotide transporter [6]). RET-mediated superoxide production responds linearly to [O_2_] in isolated mitochondria [81]. The site of RET-mediated superoxide production is disputed: some favor FMN on structural (O_2_ may be unable to access bound Q) and dielectric (superoxide anion is unlikely to migrate to the negatively charged matrix) grounds, and others claim that O_2_ could access a prosthetic semiquinone radical (SQ^●−^) during dynamic catalysis (structures are static), or it could disengage [66,69,70]. Regardless of the exact site (s), RET is occluded by compounds able to bind the Q site and/or dissipate Δ*p* [70]. 

### 2.5. Complex II

The uniquely entirely nuclear encoded and non-proton pumping complex II (i.e., succinate dehydrogenase) catalyzes succinate/fumarate and ubiquinone/ubiquinol oxidoreduction (i.e., succinate +UQ <–> fumarate + QH_2_). For many years, complex II was thought to only produce superoxide when it was damaged or mutated [82]. In 2012, Brand’s group [83] discovered that complex II can produce superoxide via its prosthetic flavin adenine nucleotide (FAD, reaction 6) moiety, in the absence of overt damage, provided key criteria are met. A flavin radical may also contribute (i.e., FAD^●^ + O_2_ –> O_2_^●−^). Specifically, succinate (forward) or ubiquinol (reverse) is required to reduce FAD, and O_2_ must be able to access FADH [69]. The redox state of the Q pool and O_2_ availability are, therefore, important determinants of complex II-mediated superoxide production. Univalent electron transfer is blocked by FAD bound dicarboxylic acids (i.e., inhibited at high (succinate)) [84]. Analogous to certain fumarate dehydrogenases [85], the redox state of the upstream iron-sulfur clusters may favor direct H_2_O_2_ production, potentially via _-_OOH release. Mathematical modelling suggests that, in the absence of respiratory inhibitors (e.g., aptenin A5 which inhibits the Q binding site of complex II), that an iron–sulfur cluster may produce superoxide (i.e., [3Fe-4S] + O_2_ –> [3Fe-4S]^−^ + O_2_^●−^) [86]. The 3Fe-4S cluster may, therefore, be a physiologically important source of complex II-derived superoxide [87]. Depending on the redox state of the complex, the 3-Fe-4S cluster and the flavin may operate to produce superoxide in parallel.

**reaction 6:** FADH^−^ + O_2_ –> FAD + O_2_^●−^

### 2.6. Complex III

**Figure 3:**
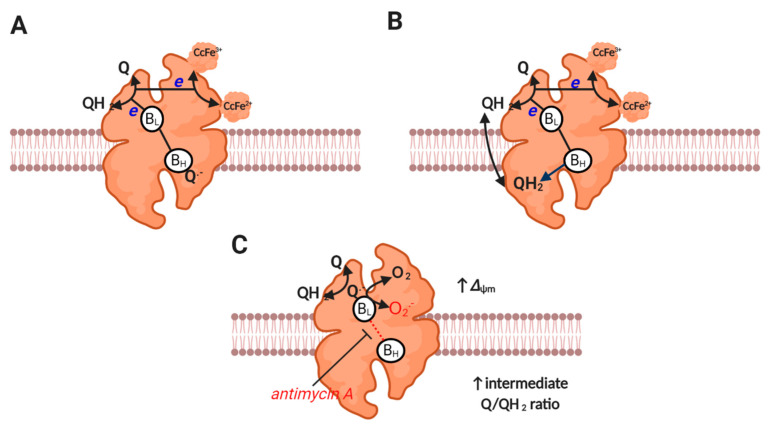
Complex III catalysis and superoxide production. (**A**) Depicts the first step of the proton motive Q cycle. UQH_2_-derived electrons bifurcate to B_L_ and Rieske protein to reduce a bound Q to Q^−^ at B_H_ and cytochrome c at cytochrome c1 reductase (both subunits are omitted for clarity). (**B**) Depicts the second step of the proton motive Q cycle wherein repeating step A results in the reduction of the bound Q to QH_2_ at B_H_. Release of QH_2_-derived protons on the P side of the membrane generates Δ*p*. Electrons tunneling between H_L_ and B_H_ do so against the prevailing Δ*p* (it is electrogenic). (**C**) Depicts superoxide production. A moderate to high ΔΨm slows electron transfer between B_L_ and B_H_. Reduced B_L_ reduces Q to Q^−^, which then reacts with O_2_ to produce superoxide (semi-reverse mechanism). Equally, a semi-forward mechanism may operate. Superoxide production seems to be favored by an intermediate Q/QH_2_ ratio (see main text).

Complex III (i.e., ubiquinol: cytochrome c oxidoreductase) is responsible for catalyzing Mitchell’s classic proton motive Q cycle [88], wherein an electron bifurcation pathway couples ubiquinol oxidation to cytochrome c reduction and proton pumping [6] (reaction 7, see Figure 3). One ubiquinol-derived electron is transferred to cytochrome c via the Reiske iron-sulfur protein and cytochrome c1, while the other electron is transferred via heme B_L_ on the P (i.e., intermembrane space), to heme B_H_ on the N (i.e., matrix) side [89]. Heme B_H_ transfers an electron to a bound ubiquinone species at the Q_i_ site to produce a stable (i.e., non-superoxide producing) SQ^●−^ intermediate that is reduced to ubiquinol when electron bifurcation is repeated. While the pioneering work of Jensen, Cadenas and others established—many decades ago—that complex III can produce superoxide [1,90,91], the actual site and mechanism is still debated [92]. Debate concerns whether a prosthetic SQ^●−^ formed near B_L_ (termed the Q_o_ site) is formed in forward mode (i.e., as a catalytic intermediate, reaction 8), or in reverse mode (i.e., a back reaction between heme B_L_ and ubiquinone, reaction 9) [92]. Much will depend on whether the electron bifurcation pathway is sequential (permits forward or reverse) or concerted (prohibits forward). Whether HO_2_^●^ is produced is unclear [93,94,95], but has implications for direct diffusion, given its uncharged nature from the P side to the matrix (i.e., site topology [96]). Superoxide directly released to the intermembrane space could diffuse to the matrix secondary to H_2_O_2_ production (mediated by HO_2_^●^ or CuZnSOD), and may be favored by deeply folded cristae [92]. Obligate complex III dimers may also reduce superoxide production by permitting electron transfer between monomers [97]. Far from being trivialities, the mechanisms are important for discerning the factors that control superoxide production (e.g., ubiquinone would promote superoxide production in reverse mode when Heme B_L_ is reduced) [98] and regulate function (e.g., HO_2_^●^ within the inner membrane could abstract a proton from a methylene group to initiate lipid peroxidation by producing a chain propagating peroxyl radical [99,100]). A better understanding of enzyme catalysis (e.g., sequential or concerted) is required to dissect how complex III produces superoxide.

**reaction 7:** Ubiquinol + 2 cytochrome c Fe(III) + 2 H+ (matrix) –> ubiquinone + ubiquinol + 2 cytochrome c Fe^2+^ + 4 H^+^ (P side, Δ*p*)

**reaction 8:** SQ.− + O_2_ –> Q + O_2_^●−^

**reaction 9:** Heme B_L_ (red) + Q –> Heme B_L_ (ox) + SQ –> SQ.− + O_2_ –> Q + O_2_^●−^

Antimycin A induces complex III-mediated superoxide production by binding to the Qi site to prohibit electron transfer between the heme B_L_ and B_H_ [101], which favors superoxide production by increasing *t*E_RED._ Inhibiting complex IV (e.g., with cyanide) can suppress complex III-mediated superoxide production by reducing cytochrome c to levels incompatible with catalysis (i.e., little to no oxidized cytochrome c available for reaction 7) [92,102,103]. Under physiological conditions, ΔΨ_m_ can sufficiently slow electron transfer between *b*_L_ and *b*_H_, to induce superoxide production at complex III [92] (see Figure 3). Mathematical modelling reveals that complex III-mediated superoxide production is increased by a high ΔΨ_m_ (rising 4-fold as it increases from 150 to 200 mV), and is favored by a partially reduced Q pool [104]. Modelling also suggests that superoxide production is negligible at high ubiquinol levels, potentially due to restricting the availability of ubiquinone for a semi-reverse mechanism and B_H_ binding (i.e., normal catalysis). The redox state of the Q pool and ΔΨ_m_ set physiological superoxide production at complex III [92]. 

### 2.7. Key Superoxide Production Modes

Four key modes of mitochondrial superoxide production exist (see Table 2). To qualify the terms high and low, a parameter (e.g., respiration) can dynamically occupy a granular modifiable (e.g., complex IV content can be increased) spectrum of allowed values between extremes. What constitutes a high and low value varies. Further, the relevant thresholds are insufficiently understood. For example, the threshold of low respiration required to induce superoxide cannot be stated absolutely, because it depends on several factors (e.g., substrate (s) oxidized). Assuming it could, [superoxide] produced would vary, potentially considerably (e.g., in mutated mitochondria), and would evolve over time (e.g., with substrate supply and ATP demand). Mode 1 is defined by comparatively low superoxide production, because OXPHOS and respiration are high due to ATP demand. Mode 1 occurs during intense skeletal muscle and neural activity [33,47,69,109,110]. Shorter electron residencies decrease *t*E_RED_ and superoxide production probability, even when O_2_ uptake is increased (i.e., less time is available for the reaction to occur). While the overall tendency is for decreased net superoxide production, some sites, particularly complex I in forward mode, can still produce superoxide at an appreciable rate, due to continued NADH availability [109].

In mode 2, high [NADH] can drive superoxide production at complex I via forward electron transfer and TCA dehydrogenases (depending on substrate availability), provided that OXPHOS/respiration are comparatively low [47,111,112]. When Δ*p* is low, a minimal role for RET and complex III would be expected. Mode 2 is sensitive to uncoupling (e.g., via uncoupling proteins [113,114,115]), because it would increase electron transfer to complex IV. Uncoupling decreases electron residency times (e.g., FMNH lifetime) to reduce the probability of superoxide production. A role for complex II could be envisaged if the reduced FAD or 3Fe-4s cluster were accessible to O_2_ [83]. In mode 3, the prevailing conditions are similar, except that a moderate to high ΔΨ_m_ drives superoxide production from complex III when the ubiquinol pool is low to moderately reduced and OXPHOS/respiration is low [92]. Mode 3 could occur alongside mode 2 when the NAD^+^/NADH pool is reduced and is sensitive to uncoupling. In mode 4, the Q pool is highly reduced, Δ*p* is near maximal, and superoxide production occurs via complex I catalyzed RET, provided OXPHOS/respiration are low [47,81].

The exact sites that operate in each mode are dependent on contextual factors, including: substrate availability, enzyme content, and several regulatory factors. For example, high ATP, as well as reversible thiol oxidation, would constrain alpha keto glutarate dehydrogenase-mediated superoxide production in mode 2 to promote complex I-mediated superoxide production [69]. In mode 4, RET would be inhibited if complex I is locked in a structural inactive state termed the D-state [72,116,117,118,119]. Different modes and sites (even within the same mode) can operate in parallel.

## 3. How Mitochondria Produce Superoxide in ART: The Unknown

### 3.1. General Considerations

Given that oocytes contain ~100,000 mitochondria and their biogenesis is repressed until the 4–8 cell stage [120], the total amount of enzyme is fixed (at least for the respiratory chain, as a turnover of TCA enzymes may be possible) at a high level. Superoxide production could, therefore, be substantial if a near maximal state of reduction (i.e., E_RED_) at 21% O_2_ were achieved. Equally, distributing electrons amongst ~100,000 mitochondria could limit E_RED_. Substrate supply, as primarily set by the media lactate pyruvate ratio [121], and OXPHOS will set E_RED_. In general, culture at 21% O_2_ (actually 18.6% in a humidified incubator with 5% CO_2_ [122]) should favor superoxide production at a given E_RED_ by increasing O_2_ by ~10–15% compared to in utero. The impact of decreasing O_2_ from 21 to 5% on mitochondrial ROS production in ART is, however, unknown. Dynamic interplay between [O_2_] and [E_RED_] means that both evolve over time with attendant implications of superoxide production, and that oxidative stress can only be rationally targeted by considering both determinants. Clinically, 5% O_2_ can improve live birth rates (+13%) [16], but its efficacy varies and is disputed [123]. Perhaps, differences in the media used and, therefore, substrate levels contributed. Suboptimal media (i.e., electron oversupply) could induce substantial mitochondrial superoxide production, even at 5% O_2_ by increasing [E_RED_]. Consistent with this, decreasing [O_2_] has, in some cases, failed to reduce ROS production [124]. Moreover, high pyruvate levels are sufficient to induce oxidative stress at a fixed O_2_ in zygotes [125]. Media changes could also induce IRI by resetting O_2_ gradients [122], with the importance of this factor for oxidative stress depending on the frequency of the media changes and whether the composition changes. That is, different substrate ratios (e.g., lactate:pyruvate ratios) could impact mitochondrial ROS production; with their effect potentially depending on the developmental stage. Additionally, the media pH and transition metal ion content can induce oxidative stress (see Section 4). The number of oocytes cultured is important because multicellularity by establishing O_2_ gradients is arguably the best defense against oxidative stress [8,126]. That is, oxidative stress should be greater in single oocytes compared to groups owing to increased [O_2_]. Additionally, light exposure induces oxidative stress due to flavin autoxidation and singlet dioxygen production in media (e.g., via riboflavin) and mitochondria (e.g., via FMN) [24,127,128,129,130].

### 3.2. Site and Mode-defined Mechanisms of Superoxide Production in ART

A popular way to infer the site of superoxide production is to treat cells/isolated mitochondria with a respiratory chain inhibitor (e.g., rotenone) to terminally arrest electron transfer at a strategic nexus, before assessing the impact on a proxy marker of superoxide production, typically a fluorescent probe (e.g., MitoSOX [131]) in vivo or ex vivo in isolated mitochondria (e.g., Amplex Red [132]). For example, if antimycin A increases the marker, then a role for complex III is assigned. That is, complex III is assumed to contribute to the superoxide production that occurs in the absence of antimycin A (i.e., the “native” rate). As Brand remarks [69,133], (1) terminally arresting electron transfer will block OXPHOS (e.g., taking mode 1 mitochondria to 2–4); (2) electron blockade will reduce upstream and oxidize downstream sites (e.g., artificially rerouting electron transfer could make an inoperative site make superoxide or vice versa); (3) certain inhibitors alter the properties of the site (e.g., antimycin A largely abolishes the effect of ΔΨ_m_ on complex III [104]); and (4) several have off-target effects (e.g., sodium azide can inhibit SOD). With the aforementioned caveats in mind, the sole study assessing the impact of respiratory chain inhibitors is judiciously critiqued. In 1991, Johnson’ group [134] showed that antimycin A and rotenone, decreased ROS levels, as inferred by cytosolic DCF fluorescence, by ~70 and 50%, respectively, at 52 h post-fertilization in murine oocytes at 21% O_2_. At the time, DCF was considered a valid H_2_O_2_ assay; but we now know that it is overtly flawed (e.g., the intermediate DCF radical can react with O_2_ to produce superoxide), and fails to appreciably react with H_2_O_2_ [135,136,137]. DCF caveats aside, their work, albeit tentatively, suggests that the Q pool—the major determinant of superoxide production at complex III under antimycin A treatment—is too oxidized or reduced to support superoxide production, which is maximal at ~50–70% Q pool reduction [104]. While chronic treatment did cause membrane blebbing [134], acute treatment with rotenone still decreased ROS levels. Their rotenone findings, again albeit with caveats, argues against a major role for forward electron transfer at the NADH linked sites (e.g., complex I). Recall, in forward mode, rotenone would be expected to approximate mode 2 by increasing [NADH]. We can, therefore, cautiously proceed on the basis that forward mode production is unlikely to play a dominant role, and the Q pool lies close to an extreme (highly oxidized or reduced), at least at the observed timepoint. Recontextualizing the original finding suggests that the unexpected inability of antimycin A and rotenone to increase ROS could reflect nuances in Q pool dependence and RET, respectively.

**Figure 4:**
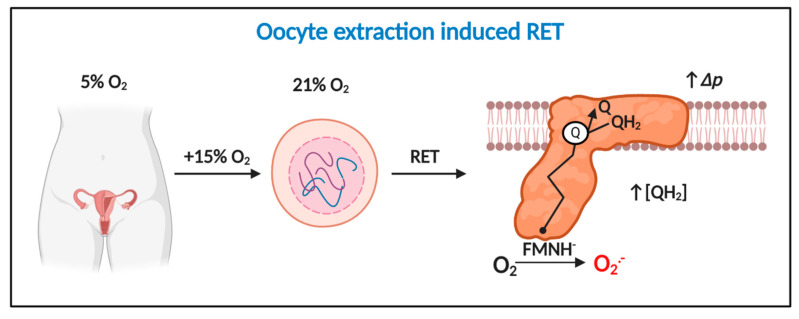
Oocyte extraction-induced RET is a potential temporally defined mechanism of mitochondrial superoxide production in assisted reproduction technologies (ART). Extracting oocytes induces a 15% increase in O_2_ exposure, which drives considerable RET-dependent O_2_^●−^ production provided Δ*p* is near maximal and QH_2_ is abundant. Extraction-induced light and damage could potentiate RET.

When Johnson’s group performed their pioneering study [134], RET was considered a physiologically irrelevant curiosity of isolated mitochondria. Murphy’s seminal work [73] suggests the ability of rotenone to sterically occlude ubiquinol oxidation-curtailed RET [70]. That is, zygote mitochondria may have been making superoxide via RET. If so, Δ*p* must be near maximal and the Q pool must be highly reduced [47]. Two findings strengthen the appeal of RET as a superoxide production mechanism. First, RET linearly responds to O_2_ [81]; which helps tie the ability of 21% O_2_ to cause oxidative stress to a discrete mechanism (Figure 4). Second, oocyte/zygotes are known to contain a distinct pool of mitochondria with a high ΔΨ_m_ [138,139,140], which may enable them to support RET. Intriguingly, loss of highly polarized mitochondria impairs cell division, implying a potential regulatory role [141,142]. However, some studies find no evidence for pools of highly polarized mitochondria [143]. Discrepancies could reflect the species, timepoint, and/or probe used [143]. The plausibility of RET as an underlying mechanism rests on the redox state of the Q pool; which is unknown, in part, because it is difficult to assess (e.g., extracted ubiquinol rapidly oxidizes to ubiquinone [144]). If RET operates, then how did antimycin A reduce ROS production? Perhaps, antimycin A dissipated Δ*p* secondary to arresting complex III and IV activity. RET may also be transiently abolished by fertilization-induced Δ*p*-dependent Ca^2+^ uptake [145]. Conversely, RET could be enhanced by a nitric oxide (NO^●^)-dependent decrease in OXPHOS due to complex IV inhibition and a resultant increase in Δ*p* [146,147]; the presence of cumulus cells is likely key to NO^●^ dependent affects [148] as is the prevailing [O_2_] (NO^●^ will exert a greater negative affect at lower [O_2_] levels). From a clinical perspective, significant RET catalyzed superoxide production can occur at comparatively low [O_2_] (e.g., ~40 nmol H_2_O_2_/min^−1^/mg^−1^ at 25 µM O_2_) [81]. That is, RET is still possible, especially at media pyruvate levels, when O_2_ is reduced from 21 to 5%.

Resolving the redox state of the Q pool is essential for appraising the veracity of an alternate possibility: rotenone by blocking ubiquinol production and decreasing Δ*p* secondary to arresting complex I activity could have decreased complex III-mediated superoxide production. Reports of highly polarized mitochondria are also, therefore, relevant to complex III (i.e., mode 3). Additionally, the ability of antimycin A or rotenone to impact the levels of dicarboxylic acids may have attenuated complex II-mediated superoxide production. In discerning between the sites and modes experiments with uncouplers (to decrease Δ*p*), malonate (sterically occlude the complex II FAD) and aptenin 5A (prevents ubiquinol driven superoxide production at complex II) may be informative [69,83,149]. For example, RET would be highly sensitive to uncouplers and malonate (to block ubiquinol production). To disambiguate the exact site (s), then selective inhibitors of mitochondrial superoxide production at complex I and complex III termed S1QEL and S3QEL, respectively, would be useful [150,151,152,153] (see Box 2). Unlike respiratory chain inhibitors (e.g., rotenone), S1QEL/S3QEL seem to selectively block superoxide production without terminally arresting electron transfer, which could make it possible to infer native sites. Disambiguating how the SQEL family inhibit superoxide production is required to interpret their effects [154].

Box 2 **Experimental tools to help advance current knowledge.**

**Mitochondria-targeted antioxidants**: Several mitochondria-targeted antioxidants are available to interrogate the importance of mitochondrial oxidative stress by manipulating the matrix redox environment [155,156]. Promising compounds include mitochondria-targeted vitamin E, vitamin C, and Q [157,158,159,160,161,162,163]. Redox-active compounds based on SOD (termed manganese porphyrins) with pleiotropic in vivo redox chemistry may prove useful [164,165,166]. Inherent chemical limits (e.g., inability to react with H_2_O_2_) mean that the failure of a single compound to improve ART cannot be taken as evidence against a role for oxidative stress: such a result is equally compatible with the inability of the compound to react with the relevant species [167]. For example, MitoVE would be relatively ineffective at attenuating matrix superoxide production (e.g., at the complex I flavin), because it concentrates in membranes and is unable to appreciably react with aqueous superoxide [168,169].

**S1QEL and S3QEL**: As discussed in text, S1QEL and S3QEL, when combined with an appropriate mitochondria-targeted probe, may prove useful for dissecting the site and mode of mitochondrial superoxide in an ART context.

**Mitochondria-targeted probes**: The ART literature is plagued by the use of flawed probes like DCF [136]. While expressing genetically encoded ratio-metric redox indicators may be problematic [170], several chemically well-understood next-generation small molecule ratio-metric probes can be used to measure mitochondrial superoxide and H_2_O_2_ (e.g., MitoNeoD and MitoB) [171,172,173]. If MitoSOX is used, it is important to assess the diagnostic superoxide specific product (2-OH-E) by HPLC [174].  

**Protein thiol oxidation**: Redox proteomics approaches are useful to measure protein thiol oxidation in a systematic high-throughput manner [175,176,177,178,179]. Immunological techniques (e.g., Click PEGylation) can also be used to assess the redox state of a protein of interest by Western blotting [180,181,182,183].

**Other**: Ratio-metric mass spectrometry-based tools exist to measure the ΔΨ_m_ and the redox state of the Q pool [184,185]. Fluorescent approaches (e.g., TMRE) can also be used to measure ΔΨ_m_ [6]. Moreover, mitochondria-targeted pro-oxidants like mitochondria-targeted paraquat and CDNB may be useful for appraising the role of complex I-derived superoxide and thiol oxidation, respectively [186,187]. Additionally, several well-established methods exist to measure mitochondrial antioxidant defense parameters (e.g., glutathione and MnSOD) [188,189].

Reports of spatially segregated pools of highly and moderately polarized mitochondria [138], together with a fertilization-induced increase in OXPHOS (i.e., sensitivity of respiration to oligomycin [125,145,190]), are consistent with some mitochondria operating in mode 1 [191,192] (with mode 1 becoming more important in the blastocyst stage). If so, then complex I (forward mode) may also make a low but persistent contribution to total superoxide production in mode 1 mitochondria, provided that the results obtained with skeletal muscle mitochondria in a metabolic milieu mimicking exercise hold in the oocyte/zygote [109]. Given the structural differences between somatic and embryonic mitochondria, and the potential functional immaturity of the latter (see [193]), further research is required to determine whether the same mechanisms translate to oocytes/zygotes. To give a recent structural example, entrapment of some of the Q pool in supercomplexes in hypoxia supports Na^+^ regulated complex III-mediated superoxide production in somatic mitochondria [194]. Clearly, the structural immaturity of embryonic mitochondria (e.g., spherical morphology) could have profound consequences for superoxide production (e.g., a lack of supercomplexes may obviate complex III-mediated superoxide production in utero, due to homogenous Q pool distribution). Our understanding of how oocyte/zygote mitochondria produce ROS is, therefore, intimately tied to mitochondrial form and function.

Given the well-documented reliance of oocyte mitochondria on pyruvate [195,196], pyruvate dehydrogenase (PDH) would be expected to contribute to superoxide production in modes 1–3. Proximal superoxide production may be self-limiting, since it can inactivate PDH via the reversible thiol oxidation of the E2 subunit [105,197]. Likewise, redox regulation of the mitochondrial pyruvate carrier may constrain pyruvate uptake [198], albeit at the expense of OXPHOS, until it can be reduced by matrix antioxidant defense. Such oscillatory behavior may support cyclic superoxide fluxes. If PDH is cyclically inactivated, then the ability of mitochondria to support lactate and amino acid metabolism, particularly via the malate-aspartate shuttle, will be key [199]. A fascinating finding arguing against a role for PDH is that it migrates to the nucleus, along with several other TCA enzymes, to support epigenetic wiring and genome activation in mouse and human zygotes [200,201]. Their ability to migrate to the nucleus reinforces the inability of rotenone to stimulate forward mode production, potentially by limiting [NADH]. A stalled TCA cycle could increase Δ*p* and [ubiquinol] to levels compatible with RET, as the rotenone finding suggests, in a significant subset of mitochondria.

### 3.3. How Oocyte/Zygote Mitochondria Produce Superoxide is Unknown

**Figure 5:**
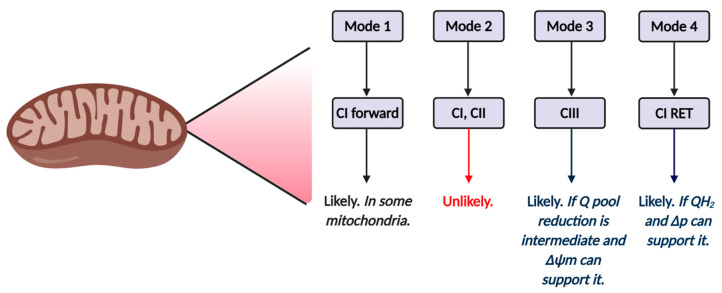
Tentative mechanisms of mitochondrial reactive oxygen species (ROS) production in ART by mode and site. Mode 1 (see Table 2) is likely to operate in at least some mitochondria, and is likely driven by complex I forward electron transfer. It is possible that complex II also operates in mode 1. Inhibitor studies (see main text) reveal forward mode is unlikely to operate in mode 2, since rotenone fails to increase ROS production in zygotes. The relative importance of mode 3 and 4 will depend on Δ*p* and the redox state of the Q pool. Inhibitor studies (see main text) support a role for RET.

Further research using next-generation tools (see Box 2) to expand the current boundary of knowledge (see Figure 5) is required because the use of flawed probes and approaches means that how oocyte/zygote mitochondria produce superoxide, both in utero and in ART, is unknown. For example, ambiguity surrounds RET as a plausible superoxide production mechanism in ART, because the redox state of the Q pool is unknown and rotenone could have had off-target effects (see Section 3.3).

Despite how oocyte/zygote mitochondria produce ROS being a known unknown, it is possible to help advance the field, because several general points will apply, regardless of the mechanism. First, superoxide production will change over time as O_2_ and E_RED_ evolve; with the latter being sensitive to substrate supply (i.e., media formula and changes). Second, superoxide production will exhibit mode and site heterogeneity within and between mitochondria (potentially at different developmental timepoints). Third, oxidative damage could impact superoxide production (see Section 4). Fourth, superoxide production is likely to vary according to the species (e.g., the reliance of porcine oocytes on beta oxidation [202] will profoundly impact the nature of the superoxide production observed). What is more, the nature of mitochondrial superoxide production will differ from what occurs in utero (i.e., the prevailing O_2_ sets a lower potential superoxide production limit in vivo). Furthermore, the known structural immaturity of oocyte and early zygotic mitochondria (e.g., spherical morphology) could impact superoxide production [203] (see Section 3.3). Finally, several timepoints, including: initial extraction; media changes, fertilization, and the metabolic switch to aerobic glycolysis will be of interest [204,205]. It would be unwise to extrapolate findings from one timepoint to another (e.g., as the PDH example in Section 3.3 attests).

## 4. A Framework for Interpreting the Role of Mitochondrial ROS in ART: The Intriguing

### 4.1. Interpreting Mitochondrial Superoxide Production

Mitochondrial superoxide production is often viewed as an unwanted side-reaction usually referred to as a “leak”, which broadly approximates a short circuit in an electrical analogy. Within complex IV, bound O_2_ is very likely reduced to superoxide (the O-O bond is weaker in superoxide compared to O_2_ [5]) via electron dissociation from the heme to O_2_, before it is concertedly reduced to H_2_O via bound O^-2^ intermediates [206]. The potential intermediacy of a transient bound superoxide to mitochondrial respiration, may make the release of superoxide via defined off-target pathways at complex I, II and III an evolutionary success as opposed to failure [207]. A radical off-pathway reporting the process of electrons—the simplest free radical—being transferred to a diradical to produce H_2_O is appealing. Deliberate superoxide production off-pathways, may enable reaction 3 to report on reactions 1 and 2 (i.e., a minor pathway reporting a major one [208]). Exquisite sensitivity to key parameters of OXPHOS, substrate supply and respiration (e.g., Δ*p*) could enable superoxide production to report mitochondrial function [81,207,209,210,211]. For example, RET responds to OXPHOS (i.e., Δ*p*), substrate supply (ubiquinol) and O_2_, making it a plausible function linked redox signal [70,81]. In support, Allen [211] contends that mitochondria retain a genome to respond to respiratory chain-derived redox signals. Basic redox chemistry means deliberate and adventitious can co-exist. For example, superoxide emanating from a deliberate pathway (e.g., FMN at complex) could lead, via hydrogen peroxide and transition metal ions, to adventitious production via hydroxyl radical-mediated attack of glutathione to a radical, which then adventitiously produces superoxide via the Winterbourn sink pathway. Regardless of whether mitochondrial superoxide production is adventitious (leak) or intended (deliberate off-pathway), it is wise to interpret superoxide production using a context-dependent functionality framework. Biological context reconciles the fundamental duality at the heart of redox biology, by determining whether the net effect of superoxide production benefits or harms the cell. From a therapeutic perspective, context-dependent functionality means that it would be unwise to abolish superoxide production in ART (i.e., it could negate an adaptive response). Consistent with this, in the presence of cumulus cells, the short-term exposure of bovine oocytes to even supraphysiological levels of H_2_O_2_ (50–100 µM, as opposed to 1–100 nM) improved subsequent zygotic development [212].

### 4.2. A Two-Step Bifurcation Model to Interpret ART-induced Oxidative Stress

**Figure 6:**
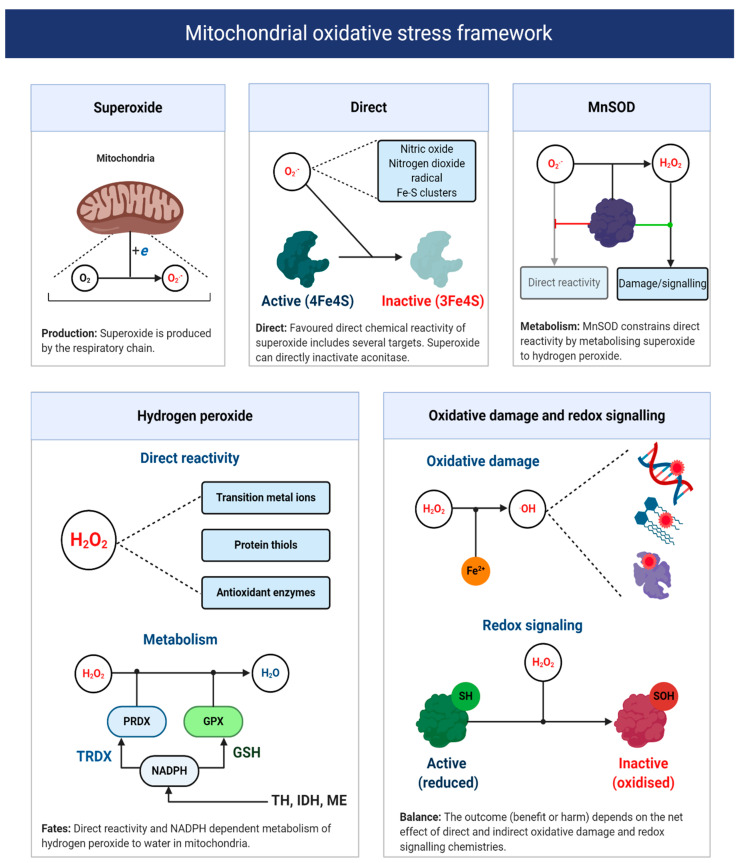
A mitochondrial oxidative stress framework. Upper panels depict the direct chemical reactivity of superoxide, which is regulated by MnSOD activity. The lower left panel depicts the direct chemical reactivity and metabolism of H_2_O_2_. The lower right panel illustrates two H_2_O_2_-focused chemical defined examples of oxidative damage and redox signaling. The net function of mitochondrial superoxide production will depend on whether direct and indirect reactivity (via H_2_O_2_ and other species like hydroxyl radical) is beneficial or harmful. Abbreviations: TH = transhydrogenase; IDH = NADPH-dependent isocitrate dehydrogenase; and ME = NADPH-dependent malic enzyme.

As Sies remarks [34], the term oxidative stress conveys no useful mechanistic information unless the underlying chemistry is defined. Recall one is unable to target ROS—no molecule called ROS exists [58]. A fragmentary understanding of ART-induced oxidative stress calls for a chemically defined two-step bifurcation model for ART-induced mitochondrial oxidative stress (see Figure 6). The first bifurcation stipulates that the nature of the oxidative stress observed depends, in part, on the ability of superoxide to evade MnSOD to directly react with its matrix interactome [208,213]. Specifically, matrix NO^●^ to produce peroxynitrite (and subsequently free radicals, reactions 10–11), protein bound (e.g., aconitase) or “free” transition metal ions [214,215,216]. The alkaline pH of the mitochondrial matrix means that MnSOD controls the direct reactivity of superoxide, because uncatalyzed dismutation (reaction 12) proceeds at a negligible rate without HO_2_^●^ (reaction 13), because two anions electrostatically repel one another [55,56,217,218,219]. HO_2_^●^ is still relevant, because it can occur as a locally caged radical when a substrate protonates superoxide [5,95]. Active and abundant MnSOD will constrain superoxide to picomolar levels, to limit direct reactivity by catalyzing reaction 12 at a diffusion controlled rate (*k*~2 × 10^9^ M^−1^ s^−1^) [220]; provided that it contains manganese as the iron ligated enzyme can produce ^●^OH [221]. As Imlay [220] calculates, even picomolar (10^−11/12^ M) levels of superoxide are sufficient to inactivate enzymes iron-sulfur proteins like aconitase, with a half-time of ~20 min in *E. coli*. By controlling the fate of superoxide, MnSOD exerts considerable control over the type of oxidative stress experienced [222], especially since, in the absence of an anion channel, superoxide will be retained in the matrix [223]. (reaction 11 in 33% yield [224,225,226]).

**reaction 10:** O_2_^●−^ + NO^●^ → ONOO^−^

**reaction 11:** ONOO^−^ + CO_2_ → ONOOCO_2_→ CO_3_^−^ + NO_2_^●^

**reaction 12:** O_2_^●−^ + O_2_^●−^ + 2H^+^ → H_2_O_2_ + O_2_

**reaction 13:** HO_2_^●^ + O_2_^●−^ +H^+^ → H_2_O_2_+ O_2_

The second bifurcation stipulates that the nature of the oxidative stress experienced depends on the partitioning of H_2_O_2_ between matrix peroxidases or other direct targets; most notably, transition metal ions and thiols. Matrix peroxidase metabolism (principally peroxiredoxin 3, 5, and glutathione peroxidase 1 [227]) is coupled to antioxidant defense, wherein H_2_O_2_ is converted to H_2_O in a process that oxidizes thioredoxin or glutathione—the reduced forms are then regenerated at the expense of NADPH by mitochondrial thioredoxin reductase and glutathione reductase, respectively [228]. NADPH can be produced by isocitrate dehydrogenase, malic enzyme, one carbon metabolism or the Δ*p* consuming transhydrogenase (e.g., NADH + NADP^+^ + H^+^ (P side) –>NAD^+^ + NADPH + H^+^ (N side) [229]. NADPH-supported redox systems could couple the excessive production of H_2_O_2_ to antioxidant defense [228]. It could also be coupled to redox signaling (discussed below) [230]. Conversely, defective antioxidant defense will favor mitochondrial H_2_O_2_ release [231] or reactivity with transition metal ions to produce ^●^OH (reaction 14) [232]. Knowledge of oocyte/early zygote antioxidant defense is limited, so their capacity to rebuff an ART-induced increase in H_2_O_2_ is unclear. In zygotes, the migration of IDH to the nucleus could limit NADPH availability [200]. The attendant consequences for oxidative damage, given the diffusion controlled reactivity of ^●^OH (*k* ~ 10^9^ M^−1^ s^−1^), are significant [233]. In particular, ^●^OH can impede OXPHOS by inactivating complex I–V [234] and cause mitochondrial DNA (mtDNA) heteroplasmy [235] by abstracting, adding and oxidizing nucleotides, notably guanine [236,237,238]. The need to maintain mtDNA homoplasmy to avoid bioenergetic defects and potentially increase superoxide production by a mismatched respiratory chain, is difficult to reconcile with active OXPHOS because, even in mode 1, it would sensitize oocytes/zygotes to DNA damage [193,209,239,240]. Perhaps, low [O_2_], together with cumulus cell-derived ATP supply limits OXPHOS in utero to protect mtDNA homoplasmy and/or any damage maintained, is repaired or prevented by DNA binding proteins. Spatial differences may segregate much of the damage into trophectoderm mitochondria, destined to become the placenta, to protect the inner cell mass.

**reaction 14:** H_2_O_2_+ Cu^+^ or Fe^2+^ –> OH + ^−^OH + Cu^2+^ or Fe(III)

Redox signaling refers to the ability of ROS to signal changing protein activity, function, lifetime, binding partners, and location [241,242,243]. While redox signals can take many guises (e.g., direct reactivity of superoxide with aconitase [208,244]), a major form is defined by H_2_O_2_-mediated reversible oxidation of protein thiols (cysteine residues) and the resultant formation of distinct chemotypes from sulfenic acids (RSOH) to disulfide bonds (RSSR), depending on contextual factors (e.g., RSOH stability [58,241,245,246]). The mammalian proteome contains over 200,000 protein thiols, with many being located in the matrix (their matrix abundance is in the mM range), often in a deprotonated state (favoring direct H_2_O_2_ reactivity), due to the alkaline pH [228,247,248,249]. I propose that: ART-induced oxidative stress is causally linked to a pervasive H_2_O_2_-mediated increase in fractional protein thiol oxidation occupancies from adaptive (physiological) to maladaptive (pathological) levels. Reversible thiol oxidation can impact superoxide production directly, by altering respiratory chain activity [250]. For example, oxidizing thiols in subunits (e.g., NDUFS1) proximal to FMN in complex I can decrease superoxide production by sterically impeding NADH access [251]; which would curtail complex I-mediated superoxide production at the expense of OXPHOS (i.e., a negative feedback loop). Reversible oxidation of the alpha subunit of complex V can impact superoxide production indirectly by altering OXPHOS [252,253,254]. Impaired complex V activity could increase Δ*p* to favor superoxide production, by reducing upstream complexes (i.e., ↑ [E_RED_]), which could promote RET or complex III-mediated superoxide production (i.e., a feedforward loop). Oxidative stress-induced defects in OXPHOS could impair ART by reducing the [ATP] available to support spindle formation, ion homeostasis, and protein synthesis [255].

Kinetically, how reversible thiol oxidation proceeds is unclear, because H_2_O_2_ reacts at a slow rate (*k*~1–50 M^−1^ s^−1^) with most thiols compared to antioxidant enzymes (*k*~10^7−8^ M^−1^ s^−1^) [256]. Several solutions to the kinetic conundrum have been proposed [257,258,259,260]. Redox relays represent an elegant solution wherein antioxidant enzymes transduce redox signals by transferring H_2_O_2_-derived electrons to target proteins (i.e., uncoupling peroxiredoxin 3 from thioredoxin 2). From a conceptual perspective, redox relays challenge the traditional view of antioxidant enzymes: they play a more nuanced role than merely removing ROS [222,230,258,261]. As Winterbourn enunciates [262], several parallel mechanisms may contribute (e.g., local inactivation of peroxiredoxins or locally caging H_2_O_2_ and the target). One ART relevant mechanism concerns bicarbonate (CO_2_/HCO_3_^−^), given its presence at relatively high levels (mM range) in oocyte/embryo media [121]. The equilibrium between H_2_O_2_ and HCO_3_^−^ yields peroxomonocarbonate (HCO_4_^−^). HCO_4_^−^ can react with protein thiols at a faster rate (sometimes a thousand times faster) than H_2_O_2_, and can be important for protein thiol oxidation (e.g., for protein tyrosine phosphatase 1B) in intact cells [263]. HCO_4_^−^ could also yield carbonate radical, which can react with thiols (*k*~4.6 × 10^7^ M^−1^ s^−1^) secondary to reacting with Cu^+^ (reaction 15). Another relevant possibility involves local thiol oxidation secondary to H_2_O_2_, reacting with a protein bound transition ion (e.g., Cu^+^) to produce ^●^OH; which could yield a thiyl radical (RS^●^) at a significant rate (*k*~7 × 10^9^ M^−1^ s^−1^ for L-Cys) (reaction 16). ONOO^−^-derived radicals may also contribute [228,264]. Thiyl radicals react appreciably with NO^●^ to form RSNO (reaction 17), or with another thiol (reaction 18) to yield a highly reducing radical disulfide (RSSR^●^). RSSR^●^ reacts with O_2_ at an appreciable rate (*k*~5 × 10^8^ M^−1^ s^−1^ for glutathione disulfide radical), to form superoxide (reaction 19), making it possible, in principle, for any protein thiol to produce superoxide [265]. RSSR^●^ could be an adventitious source of superoxide in mitochondria (see Section 4.1 and footnote 3). MnSOD activity to remove superoxide is, therefore, important [266]. A role for free radicals in thiol-based redox signaling has been proposed [267] and evidenced for complex I [268].

**reaction 15:** HCO_4_^−^ + Cu^+^ –> CO_3_^−^ + Cu^2+^ + OH

**reaction 16:** OH + RSH –> RS^●^ + H_2_O

**reaction 17:** RS + NO –> RSNO

**reaction 18:** RS + RSH –> RSSR^●^ + H^+^

**reaction 19:** RSSR + O_2_ –> O_2_^●−^ + RSSR

**Figure 7:**
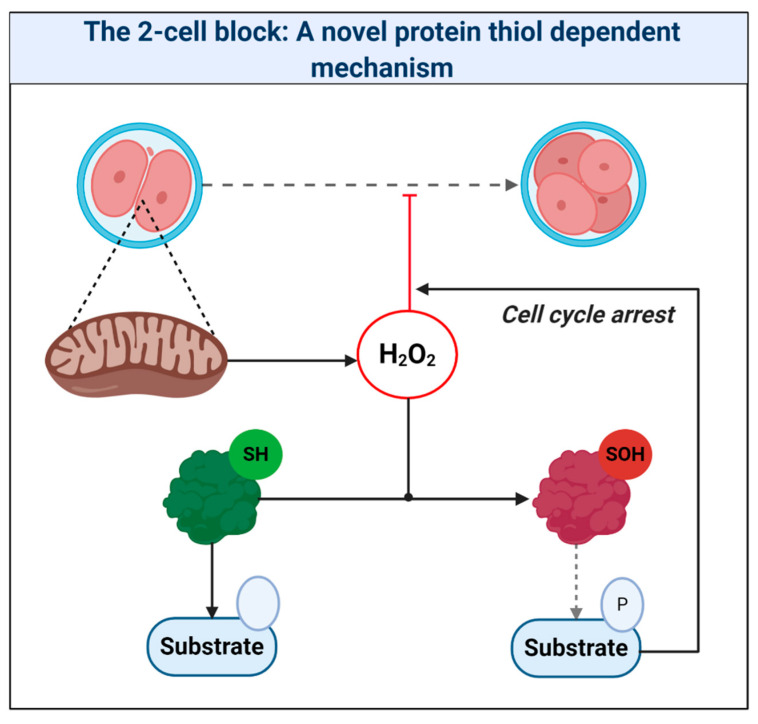
The 2-cell block: A novel protein thiol-dependent mechanism. The scheme depicts mitochondria-derived hydrogen peroxide oxidizing the catalytic (Cys373) thiol of the tyrosine protein phosphatase cdc25c to a sulfenic acid (SOH). Thiol oxidation renders cdc25c unable to dephosphorylate target substrates. The resultant perturbation in zygotic phosphorylation tone could cause cell cycle arrest.

A pervasive increase in thiol oxidation is a novel cause of oxidative stress in ART. The mechanism reconciles the ability of the thiol oxidant diamide to disrupt OXPHOS in ART [269], and reductants like 1-4-dithiothreitol to improve ART [11]. The novel framework presented should provide a useful paradigm to interpret mitochondrial oxidative stress in ART. Research is, however, required to unravel the chemical nature of the oxidative stress and its relationship to proximal mitochondrial superoxide production. It is vital to understand the physiological role of any intrinsic fertilization-induced increase in ROS production [270]. While fertilization is well known to increase H_2_O_2_ production in sea urchins [271,272,273], Amaya’s group [274] recently tied fertilization triggered Ca^2+^-induced mitochondrial ROS release to the regulation of the embryonic cell cycle via reversible cdc25c oxidation in *Xenopus laevis*. By tying mitochondrial ROS to the early cell cycle via thiol oxidation, their work provides a precedent to reimagine the 2-cell block as increased thiol oxidation of regulatory cell cycle proteins [183] (see Figure 7). Consistent with this, reversing protein thiol oxidation may explain the ability of recombinant thioredoxin to release the 2-cell block [22,261,275,276]. To disambiguate the molecular details of ART-induced oxidative stress (e.g., source-target relationships), protein thiol oxidation of key regulatory proteins should be assessed [183,277]. Finally, intimate ties between mitochondria-regulated metabolites (e.g., succinate) and epigenetics raise the potential for far-reaching ART-induced oxidative stress, secondary to aberrant epigenetic wiring [278,279,280], which could impact imprinting [281]. Precedent exists: stochastic development fluxes in the redox state of the glutathione pool regulate lifespan and stress resistance in *C. elegans* [282,283]. Moreover, the enzymes responsible were regulated by reversible thiol oxidation [282]. In mammals, embryonic exposure to rotenone, which modifies complex I-mediated superoxide production, can produce lasting DNA methylation changes in offspring [284]. Future studies may wish to interrogate the potential regulatory role of ROS-mediated retrograde mito-nuclear epigenetic signaling [285].

## 5. Conclusions

The known importance of ART-induced oxidative stress belies how oocyte/zygote mitochondria produce ROS being the unknown. Despite topical interest, current understanding of how oocyte/zygote mitochondria produce superoxide is unsatisfactory owing to the use of outmoded approaches (e.g., respiratory poisons) and methodologies (e.g., flawed probes). Considering how somatic mitochondria produce superoxide (i.e., the known [47]) makes several tractable and experimentally testable predictions about the unknown. For example, oocyte extraction may induce RET, owing to at least a 10% increase in O_2_ exposure, provided that Δ*p* is near maximal and QH_2_ is abundant. Further research using next-generation tools is required to unequivocally dissect how oocyte/zygotic mitochondria produce ROS, with a view to understanding their likely nuanced biological roles. While some of the ROS produced by oocyte/zygote mitochondria will undoubtedly cause oxidative damage [228], their ability to transduce beneficial redox signals (e.g., to report OXPHOS [209]) raises the intriguing possibility that they regulate key developmental processes (e.g., the cell cycle). Irrespective of whether the net effect of mitochondria producing ROS is useful, neutral, or harmful, plausible explanatory source-target relationships are lacking. To address this unmet need, a chemically defined oxidative stress framework is presented. The intriguing possibility of redox signaling allied to several discrete sites and modes of superoxide production means that it has arguably never been more essential to understand the mechanistic basis of ART-induced oxidative stress to rationally target key enzymes/processes to preserve redox signaling while attenuating oxidative damage.

## Figures and Tables

**Table 1:** Additional sites of superoxide production in mitochondria by enzyme, substrate, iso-potential group, source and topology. Commentary is provided as appropriate.

Enzyme	Substrate	Group	Source/Topology	Comments
Alpha keto glutarate dehydrogenase	Alpha keto glutarate.	NAD^+^/NADH	FAD of E3 dihydrolipoamide dehydrogenase. Matrix.	ROS production is inhibited by high ATP and aspartate [69] and regulated by reversible thiol oxidation [105]. Favored by high NAD^+^/NADH ratio. May also produce H_2_O_2_. Requires substrate. Ca^2+^ sensitive.
Pyruvate dehydrogenase	Pyruvate.	NAD^+^/NADH	FAD of E3 dihydrolipoamide dehydrogenase. Matrix.	Regulated by reversible thiol oxidation [105] and phosphorylation. ROS production is favored by high NAD^+^/ NADH ratio. May also produce H_2_O_2._ Requires substrate.
Branched-chain 2-oxoacid dehydrogenase complex	Branched chain 2-oxoacids (e.g., 3-methyl-2-oxopentanoate).	NAD^+^/NADH	FAD of E3 dihydrolipoamide dehydrogenase. Matrix	Favored by high NAD^+^/NADH ratio. Requires substrate.
Aminoadipate dehydrogenase complex	2-oxoadipate.	NAD^+^/NADH	FAD of E3 dihydrolipoamide dehydrogenase. Matrix	ROS production is favored by high NADH/NAD^+^ ratio. Requires substrate.
*sn*-glycerol-3-phosphate dehydrogenase	Glycerol 3-phosphate	UQ/UQH_2_	UQ binding site but may also involve a flavin. Intermembrane space and matrix.	Ca^2+^ sensitive—can enhance ROS production at low substrate levels [106]. Requires substrate. Much emanates from complex II.
Dihydroorotate dehydrogenase	Dihydroorotate	UQ/UQH_2_	UQ binding site. Matrix.	Relatively low superoxide producing capacity but can drive other sites to high rates by reducing the Q pool [107].
Electron transferring-flavoprotein: ubiquinone oxidoreductase	Electron transferring flavoprotein (involved in lipid + amino acid metabolism).	UQ/UQH_2_	May emanate from the flavin but origin is unclear [69].	ROS production is quite low even when other sites are inhibited [108], suggesting main contribution under native conditions is to reduce the Q pool.

**Table 2:** Summary of the main modes of mitochondrial ROS production. The dominate electron transport chain site is listed for each mode. The site may vary according the prevailing conditions. In practice, when one considers Table 1, other sites will also operate depending on the substrates being oxidized (e.g., pyruvate dehydrogenase (PDH) in mode 2). X denotes that a given variable has no known appreciable role in governing the mode.

Mode	OXPHOS	Respiration	Δp	NADH	QH_2_	Dominant ETC Site(s)
1	High	High	Mod	Low to mod	Low	Complex I (forward)
2	Low-mod	Low-mod	X	High	Intermediate	Complex I (forward), complex II
3	Low-mod	Low-mod	Mod	X	Intermediate	Complex III, complex II
4	Negligible	Some *	High	X	High	Complex I (reverse)

* Needs some for complex III and IV to keep Δ*p* high.
